# Supplementary material for: Uric acid levels mediate the association between four dietary indices and kidney stones in US adults: A cross-sectional study of NHANES 2007–2018
Source: PLoS One. 2026 Jan 2;21(1):e0339839. doi: 10.1371/journal.pone.0339839 (PMC12758690; doi:10.1371/journal.pone.0339839)
Supplement: S1 Table — (DOCX) [file pone.0339839.s001.docx]

Table S1 Distribution of dietary indices for NHANES 2007-2018 (n = 25421).

| Dietary indices | Mean | Geometric Mean | Percentile | | | | |
| --- | --- | --- | --- | --- | --- | --- | --- |
|  |  |  | 5th | 25th | 50th | 75th | 95th |
| MED | 3.468 | 3.172 | 1.500 | 2.500 | 3.500 | 4.500 | 6.000 |
| HEI2020 | 51.530 | 50.103 | 33.079 | 42.748 | 50.757 | 59.6466 | 72.784 |
| AHEI | 38.549 | 36.769 | 20.856 | 30.268 | 37.804 | 46.068 | 58.667 |
| DASH | 22.379 | 21.814 | 15.000 | 18.500 | 22.000 | 25.500 | 31.500 |

MED, Mediterranean Diet; HEI2020, Healthy Eating Index-2020; AHEI, Alternate Healthy Eating Index; DASH, Dietary Approaches to Stop Hypertension
