# Supplementary material for: Uric acid levels mediate the association between four dietary indices and kidney stones in US adults: A cross-sectional study of NHANES 2007–2018
Source: PLoS One. 2026 Jan 2;21(1):e0339839. doi: 10.1371/journal.pone.0339839 (PMC12758690; doi:10.1371/journal.pone.0339839)
Supplement: Attachment — Submitted filename: PONE-D-25-41276_reviewer.docx [file pone.0339839.s003.docx]

PLOS ONE

**Uric acid levels mediate the association between four dietary indices and kidney stones in US adults: a cross-sectional study of NHANES 2007-2018**

--Manuscript Draft--

| **Manuscript Number:** | PONE-D-25-41276 |
| --- | --- |
| **Article Type:** | Research Article |
| **Full Title:** | Uric acid levels mediate the association between four dietary indices and kidney stones in US adults: a cross-sectional study of NHANES 2007-2018 |
| **Short Title:** | Uric acid levels mediate the association between four dietary indices and kidney stones |
| **Corresponding Author:** | jinlong cao  Xi'an Jiaotong University Second Affiliated Hospital xi‘an, CHINA |
| **Keywords:** | Kidney stone; Dietary indices; Uric acid; WQS regression; BKMR |
| **Abstract:** | Background  Diet and uric acid are closely linked to the formation of kidney stones. However, the specific dietary indices associated with kidney stone risk and the mediating role of uric acid remain unclear. This study investigates the association between four dietary indices and kidney stone risk while exploring the mediating role of uric acid.  Methods  Data were obtained from the National Health and Nutrition Examination Survey (NHANES). Four dietary indices were calculated: the Mediterranean Diet (MED), Healthy Eating Index-2020 (HEI-2020), Alternate Healthy Eating Index (AHEI), and Dietary Approaches to Stop Hypertension (DASH). Univariate logistic regression analysis and restricted cubic splines (RCS) curve were used to analyze the single effect of dietary index and kidney stones. Weighted Quantile Sum (WQS) and Bayesian Kernel Machine Regression (BKMR) to visualize the mixed effects of multiple dietary indices and kidney stone risk. Finally, mediation analyses were employed to assess associations and mediation effects.  Results  Among 25,421 participants, 2,470 had a history of kidney stones. All four dietary indices showed a significant negative association with kidney stone risk, with MED and HEI-2020 showing the strongest effects. WQS regression results indicated that the mixed effects of the four dietary indices were negatively correlated with kidney stones, with the highest weight attributed to HEI2020 (49.2%), followed by DASH (26.4%), MED (21.8%), and AHEI (2.9%). BKMR analysis revealed a negative exposure- response trend for each dietary indices to the risk of kidney stones and HEI2020, DASH, MED, and AHEI are negative related with kidney stones at all three quantiles. Mediation analysis revealed that uric acid mediated the relationship between dietary indices and kidney stone risk, with mediation proportions of 25.56% (MED), 12.14% (AHEI), 5.88% (DASH), and 2.52% (HEI-2020).  Conclusion  Healthy dietary patterns are associated with a reduced risk of kidney stones, partially mediated by uric acid levels. |
| **Order of Authors:** | jinlong cao |
|  | Tianyuan Zhai |
|  | Lingyu Guo |
|  | Yue Chong |
|  |  |

*Powered by Editorial Manager® and ProduXion Manager® from Aries Systems Corporation*

|  | Qi Chen |
| --- | --- |
|  | Qian Wang |
|  | Delai Fu |
|  | Li Xue |
|  | Feng Li |
| **Additional Information:** |  |
| **Question** | **Response** |
| **Financial Disclosure**  Enter a financial disclosure statement that describes the sources of funding for the work included in this submission. Review the [submission guidelines](http://journals.plos.org/plosone/s/submission-guidelines#loc-financial-disclosure-statement) for detailed requirements. View published research articles from [*PLOS ONE*](https://journals.plos.org/plosone/) for specific examples.  This statement is required for submission and **will appear in the published article** if the submission is accepted. Please make sure it is accurate. | Yes |
| **Funded studies**  Enter a statement with the following details:   - Initials of the authors who received each award - Grant numbers awarded to each author - The full name of each funder - URL of each funder website - Did the sponsors or funders play any role in the study design, data collection and analysis, decision to publish, or preparation of the manuscript? |  |
| Did you receive funding for this work? |  |
| Please add funding details.  as follow-up to "**Financial Disclosure**  Enter a financial disclosure statement that describes the sources of funding for the work included in this submission. Review the [submission guidelines](http://journals.plos.org/plosone/s/submission-guidelines#loc-financial-disclosure-statement) for detailed requirements. View published research articles from [*PLOS ONE*](https://journals.plos.org/plosone/) for specific examples. | This work was supported by the Natural Science Basic Research Program of Shaanxi Province (Grant No. 2020JQ-544), and the National Science Foundation for Young Scientists of China (Grant No. 82100812). |

*Powered by Editorial Manager® and ProduXion Manager® from Aries Systems Corporation*

| This statement is required for submission and **will appear in the published article** if the submission is accepted. Please make sure it is accurate. |  |
| --- | --- |
| **Funded studies**  Enter a statement with the following details:   - Initials of the authors who received each award - Grant numbers awarded to each author - The full name of each funder - URL of each funder website - Did the sponsors or funders play any role in the study design, data collection and analysis, decision to publish, or preparation of the manuscript? |  |
| Did you receive funding for this work?" |  |
| Please select the country of your main research funder (please select carefully as in some cases this is used in fee calculation).  as follow-up to "**Financial Disclosure**  Enter a financial disclosure statement that describes the sources of funding for the work included in this submission. Review the [submission guidelines](http://journals.plos.org/plosone/s/submission-guidelines#loc-financial-disclosure-statement) for detailed requirements. View published research articles from [*PLOS ONE*](https://journals.plos.org/plosone/) for specific examples.  This statement is required for submission and **will appear in the published article** if the submission is accepted. Please make sure it is accurate. | CHINA - CN |

*Powered by Editorial Manager® and ProduXion Manager® from Aries Systems Corporation*

| **Funded studies**  Enter a statement with the following details:   - Initials of the authors who received each award - Grant numbers awarded to each author - The full name of each funder - URL of each funder website - Did the sponsors or funders play any role in the study design, data collection and analysis, decision to publish, or preparation of the manuscript? |  |
| --- | --- |
| Did you receive funding for this work?" |  |
| **Competing Interests**  Use the instructions below to enter a competing interest statement for this submission. On behalf of all authors, disclose any [competing interests](http://journals.plos.org/plosone/s/competing-interests) that could be perceived to bias this work—acknowledging all financial support and any other relevant financial or non- financial competing interests.  This statement is required for submission and **will appear in the published article** if the submission is accepted. Please make sure it is accurate and that any funding sources listed in your Funding Information later in the submission form are also declared in your Financial Disclosure statement.  View published research articles from  [*PLOS ONE*](http://journals.plos.org/plosone/) for specific examples. | The authors have declared that no competing interests exist. |

*Powered by Editorial Manager® and ProduXion Manager® from Aries Systems Corporation*

| **NO authors have competing interests**  Enter: *The authors have declared that no competing interests exist.*  **Authors with competing interests**  Enter competing interest details beginning with this statement:  *I have read the journal's policy and the authors of this manuscript have the following competing interests: [insert competing interests here]* |  |
| --- | --- |
| * typeset |  |
| **Ethics Statement**  Enter an ethics statement for this submission. This statement is required if the study involved:   - Human participants - Human specimens or tissue - Vertebrate animals or cephalopods - Vertebrate embryos or tissues - Field research   Write "N/A" if the submission does not require an ethics statement.  General guidance is provided below. Consult the [submission guidelines](http://journals.plos.org/plosone/s/submission-guidelines#loc-guidelines-for-specific-study-types) for detailed instructions. **Make sure that all information entered here is included in the Methods section of the manuscript.** | N/A |

*Powered by Editorial Manager® and ProduXion Manager® from Aries Systems Corporation*

| **Format for specific study types**  **Human Subject Research (involving human participants and/or tissue)**   - Give the name of the institutional review board or ethics committee that approved the study - Include the approval number and/or a statement indicating approval of this research - Indicate the form of consent obtained (written/oral) or the reason that consent was not obtained (e.g. the data were analyzed anonymously)   **Animal Research (involving vertebrate animals, embryos or tissues)**   - Provide the name of the Institutional Animal Care and Use Committee (IACUC) or other relevant ethics board that reviewed the study protocol, and indicate whether they approved this research or granted a formal waiver of ethical approval - Include an approval number if one was obtained - If the study involved *non-human primates*, add *additional details* about animal welfare and steps taken to ameliorate suffering - If anesthesia, euthanasia, or any kind of animal sacrifice is part of the study, include briefly which substances and/or methods were applied   **Field Research**  Include the following details if this study involves the collection of plant, animal, or other materials from a natural setting:   - Field permit number - Name of the institution or relevant body that granted permission |  |
| --- | --- |
|  |  |
| **Data Availability**  Authors are required to make all data underlying the findings described fully available, without restriction, and from the time of publication. PLOS allows rare exceptions to address legal and ethical concerns. See the [PLOS Data Policy](http://journals.plos.org/plosone/s/data-availability) and [FAQ](http://journals.plos.org/plosone/s/data-availability#loc-faqs-for-data-policy) for detailed information. | Yes - all data are fully available without restriction |

*Powered by Editorial Manager® and ProduXion Manager® from Aries Systems Corporation*

| A Data Availability Statement describing where the data can be found is required at submission. Your answers to this question constitute the Data Availability Statement and **will be published in the article**, if accepted. |  |
| --- | --- |
| **Important:** Stating ‘data available on request from the author’ is not sufficient. If your data are only available upon request, select ‘No’ for the first question and explain your exceptional situation in the text box. |  |
| Do the authors confirm that all data underlying the findings described in their manuscript are fully available without restriction? |  |
| **Describe where the data may be found in full sentences. If you are copying our sample text, replace any instances of XXX with the appropriate details.**   - If the data are **held or will be held in a public repository**, include URLs, accession numbers or DOIs. If this information will only be available after acceptance, indicate this by ticking the box below. For example: *All XXX files are available from the XXX database (accession number(s) XXX, XXX.*). - If the data are all contained **within the manuscript and/or Supporting Information files**, enter the following: *All relevant data are within the manuscript and its Supporting Information files.* - If neither of these applies but you are able to provide **details of access elsewhere**, with or without limitations, please do so. For example:   *Data cannot be shared publicly because of [XXX]. Data are available from the XXX Institutional Data Access / Ethics Committee (contact via XXX) for researchers who meet the criteria for access to confidential data.*  *The data underlying the results presented in the study are available from (include the name of the third party* | All relevant data are within the manuscript and its Supporting Information files. |

*Powered by Editorial Manager® and ProduXion Manager® from Aries Systems Corporation*

| *and contact information or URL).*   - This text is appropriate if the data are owned by a third party and authors do not have permission to share the data.   * typeset |  |
| --- | --- |
| Additional data availability information: |  |

*Powered by Editorial Manager® and ProduXion Manager® from Aries Systems Corporation*

Manuscript	[Click here to access/download;Manuscript;Manuscript.docx](https://www2.cloud.editorialmanager.com/pone/download.aspx?id=41407781&guid=70eacca1-e0fd-4c7a-acb3-2e86725913cb&scheme=1)[<https://www2.cloud.editorialmanager.com/pone/download.aspx?id=41407781&guid=70eacca1-e0fd-4c7a-acb3-2e86725913cb&scheme=1>](https://www2.cloud.editorialmanager.com/pone/download.aspx?id=41407781&guid=70eacca1-e0fd-4c7a-acb3-2e86725913cb&scheme=1)

# Uric acid levels mediate the association between four dietary indices and kidney stones in US adults: a cross-sectional study of NHANES 2007-2018

Jinlong Cao^1^, Tianyuan Zhai^1^, Lingyu Guo^1^, Yue Chong^1^, Qi Chen^1^, Qian Wang^1^, Delai Fu^1^, Li Xue^1^, Feng Li^1,^ ^#^

1 Department of Urology, The Second Affiliated Hospital of Xi'an Jiaotong University, Xi'an, China.

\# Corresponding author: Feng Li, Department of Urology, The Second Affiliated Hospital of Xi’an Jiaotong University, 157 West Fifth Road, Xi’an 710000, China. Email: [lifeng.uro@xjtu.edu.cn.](mailto:lifeng.uro@xjtu.edu.cn)

# Uric acid levels mediate the association between four dietary indices and kidney stones in US adults: a cross-sectional study of NHANES 2007-2018

**Abstract**

**Background:** Diet and uric acid are closely linked to the formation of kidney stones. However, the specific dietary indices associated with kidney stone risk and the mediating role of uric acid remain unclear. This study investigates the association between four dietary indices and kidney stone risk while exploring the mediating role of uric acid.

**Methods:** Data were obtained from the National Health and Nutrition Examination Survey (NHANES). Four dietary indices were calculated: the Mediterranean Diet (MED), Healthy Eating Index-2020 (HEI-2020), Alternate Healthy Eating Index (AHEI), and Dietary Approaches to Stop Hypertension (DASH). Univariate logistic regression analysis and restricted cubic splines (RCS) curve were used to analyze the single effect of dietary index and kidney stones. Weighted Quantile Sum (WQS) and Bayesian Kernel Machine Regression (BKMR) to visualize the mixed effects of multiple dietary indices and kidney stone risk. Finally, mediation analyses were employed to assess associations and mediation effects.

**Results:** Among 25,421 participants, 2,470 had a history of kidney stones. All four dietary indices showed a significant negative association with kidney stone risk, with MED and HEI-2020 showing the strongest effects. WQS regression results indicated that the mixed effects of the four dietary indices were negatively correlated with kidney stones, with the highest weight attributed to HEI2020 (49.2 %), followed by DASH (26.4 %), MED (21.8%), and AHEI (2.9 %). BKMR analysis revealed a negative exposure-response trend for each dietary indices to the risk of kidney stones and HEI2020, DASH, MED, and AHEI are negative related with kidney stones at all three quantiles. Mediation analysis revealed that uric acid mediated the relationship between dietary indices and kidney stone risk, with mediation proportions of 25.56% (MED), 12.14% (AHEI), 5.88% (DASH), and 2.52% (HEI-2020).

**Conclusion:** Healthy dietary patterns are associated with a reduced risk of kidney stones, partially mediated by uric acid levels.

**Key words:** Kidney stone, Dietary indices, Uric acid, WQS regression, BKMR

# 1. Introduction

Kidney stone is one of the common urinary system diseases worldwide, and the prevalence is increasing worldwide. In the United States, the prevalence of kidney stones has long been around 10% in the adult population, with a higher incidence among males compared to females [1-3]. The formation of kidney stones is influenced by a variety of common factors. Underlying conditions such as obesity, gout, asthma, and diabetes can all contribute to an increased incidence of kidney stones [4-6]. Furthermore, inadequate fluid intake [7], a high-salt diet [8], diets rich in animal protein, and low- fiber dietary habits can elevate the risk of stone formation [9]. Notably, the recurrence rate of kidney stones can reach up to 50% within 5 to 10 years post-lithotripsy, posing a significant threat to individuals' health and quality of life [2, 3]. Therefore, it is important to investigate the factors associated with the occurrence of kidney stones and develop effective prevention strategies.

A healthy balanced diet is very essential to prevent kidney stones. As a tool to evaluate diet quality, dietary indices have gradually become an effective means to study the relationship between diet and various diseases [10, 11]. Previous studies have shown that higher Dietary Inflammatory Index (DII) scores and increased intake of pro- inflammatory diets are associated with an increased incidence and recurrence of kidney stones [12]. Niloofar et. al found that a higher Dietary Insulin Load (DIL) is directly related to the likelihood of kidney stones [13]. Additionally, a large cohort study in China indicated that adhering to a balanced dietary pattern, rather than a plant-based diet, is associated with a lower risk of kidney stones [14]. Above all, dietary factors play an important role in the formation of kidney stones, but the mechanisms through which different dietary patterns influence kidney stones are not fully understood. Uric acid is one of the significant factors in kidney stone formation, particularly in the process of stone formation involving components such as oxalates and calcium salts, where elevated uric acid levels may exacerbate stone deposition [15]. This indicates that the animal-based diets may mediate the occurrence of kidney stones by influencing uric acid levels, but the specific mechanisms underlying this process still lack adequate

evidence.

To our knowledge, few studies have investigated the relationship between healthy dietary indies and the formation of kidney stones. Therefore, our research aims to evaluate the association between four dietary indices (namely, the Mediterranean Diet (MED), the Healthy Eating Index-2020 (HEI2020), the Alternate Healthy Eating Index (AHEI), and the Dietary Approaches to Stop Hypertension (DASH)) and kidney stones using data from the National Health and Nutrition Examination Survey (NHANES), and to explore the mediating role of uric acid through mediation analysis. Our study uncovers potential associations between healthy dietary patterns and kidney stones, and to provide a scientific basis for the prevention and treatment of kidney stones through dietary interventions.

# 2. Methods

2.1 **Study population and design**

The raw data for this study were obtained from the NHANES III database (https://[www.cdc.gov/nchs/nhanes/),](http://www.cdc.gov/nchs/nhanes/)) with the study population and design conducted by the Centers for Disease Control and Prevention (CDC) and the National Center for Health Statistics (NCHS). The data from NHANES has been approved by the Institutional Review Board of the NCHS, and all participants provided written informed consent. We included data from six cycles (2007-2018), a total of 59,842 participants, as these cycles provided both kidney stone history and dietary and uric acid test data. Participants were excluded if they were under 20 years old and lacked kidney stone information (25,072 individuals), lacked dietary information (7,923 individuals), and lacked uric acid data (1,335 individuals). In the end, 25,421 participants were included in this study, with the detailed screening process shown in Figure 1.

# 2.2 Dietary indices

The raw data of the four dietary indices was assessed using 48-hour dietary recall data. Dietary intake data were used to estimate the types and quantities of foods and beverage consumed during the 48-hour prior to the interview and to estimate the intake of energy, nutrients, and other food components from these foods and beverages. These

data were collected through two retrospective interviews. Nutrients and energy for each food or beverage were calculated using the Food and Nutrition Database for Dietary Studies (FNDDS), The USDA Food Pattern Equivalents Database (FPED) guideline was used for food group classification. Our analysis is grounded in data from four widely recognized dietary pattern indices that serve as proxies for dietary quality: MED, HEI2020, AHEI and DASH diets. The MED is a healthy dietary pattern characterized by increased consumption of legumes, vegetables, fruits, olive oil, whole grain grains, and nuts. It also includes moderate consumption of fish and red wine, while limiting consumption of red meat products and saturated fatty acids. The HEI-2020 assesses diet quality according to the Dietary Guidelines for Americans 2020-2025 through 13 food groups or nutrient components, with a total score of 100, and higher scores indicate better diet quality. It provides a comprehensive assessment of dietary habits and the complex effects of diet on disease risk and metabolic disorders. The AHEI was extended from HEI-2010 to include more dietary factors related to chronic disease risk, based on the intake scores of 11 foods and nutrients. The DASH diet was originally developed by the National Heart, Lung, and Blood Institute (NHLBI) as a dietary model for the prevention and control of hypertension and is now considered to be one of the ideal dietary options for all adults. The four dietary indices were calculated with the use of the dietaryindex R package[16].

# 2.3 Definition of kidney stones

The Kidney Conditions - Urology questionnaire was directed at adults aged 20 years and older, which includes questions about a history of kidney stones. Survey participants who answered yes to ‘(KIQ026) Have you/Has sample person (SP) ever had a kidney stone?’ were considered to have a history of kidney stones. And no was considered to no kidney stones history. Don’t known and refused were excluded.

# 2.4 Mediation variables assessment

A standard biochemistry test was conducted by trained laboratory technicians, and uric acid concentration was measured using a timed endpoint method. Detailed instructions about analytical methodologies, principles, and operating procedures are

shown in the NHANES Laboratory Method Files.

# 2.5 Covariate assessment

Potential covariates were identified a priori based on a literature review[8, 12], including age, BMI, cotinine (substitute for smoking), gender (male/female), marital status (Divorced/ Living with partner/ Married/ Never married/ Separated/ Widowed), alcohol, race (Mexican American/ Non-Hispanic Black/ Non-Hispanic White/ Other Hispanic/ Other Race - Including Multi-Racial), education (College Graduate or above/ High School Grad/ Less Than 9th Grade/ Some College or AA degree), hypertension and Diabetes. Cotinine content, it would be more accurate to quantify the level of human exposure to tobacco smoke [17, 18]. Alcohol status was categorized into five groups: never consumption (<12 drinks in lifetime), former consumption (≥12 drinks in 1 year and did not drink last year, or did not drink last year but drank ≥12 drinks in lifetime), mild consumption (1 drinks/d for female and 2 drinks/d for male), moderate consumption (2 drinks/d for female and 3 drinks/d for male), and heavy consumption (3 drinks/d for female and 4 drinks/d for male)[19].

# 2.6 Statistical analysis

Statistical analyses were performed according to the NHANES data analysis reporting guidelines, and all analyses were performed on a weighted treatment basis. In data processing, continuous data were expressed mean ± standard deviation and analyzed by t-tests, categorical data were expressed as proportion and analyzed by χ2 test.

Binary logistic regression was applied to assess the correlation between dietary index and kidney stones. Restricted cubic spline (RCS) models were used to determine a possible dose-response relationship between dietary index and kidney stones. The weighted quantile sum (WQS) regression model explored the association between multiple dietary indices and kidney stones. The magnitude of the weights shows the contribution of each dietary index to the WQS index, with higher values indicating greater effects. Data were tested and validated using 40% and 60% stratified random samples, with 1000 bootstrap iterations[20]. Given the potential nonlinear and non-

additive relationships between dietary index scores, Bayesian kernel machine regression (BKMR) was used to visualize the association between multiple dietary index scores and kidney stone risk [21]. Finally, we evaluated the mediation effect of four dietary indices on the effect of kidney stones through uric acid.

R (version 4.2.1) was used for all statistical analyses, and P < 0.05 (two-side test) were considered to statistical significance. WQS was performed using the gWQS package, BKMR was performed using the BKMR package, and mediation effect analysis was performed using the Mediation package.

# 3. Results

3.1 **Baseline characteristics of the participants**

This cross-sectional study included 25421 participants, of whom 2470 had kidney stones and 22951 had no kidney stones. Table 1 shows the baseline characteristics of the enrolled participants. Patients with kidney stones were older, more likely to be male, and had a higher BMI than patients without stones. The proportion of hypertension and diabetes in the kidney stone group was also higher (P < 0.05). The smoking index cotinine indicates that smoking promotes the occurrence of kidney stones. In addition, education level and marital status also had a certain impact on kidney stones. For the MED, HEI2020, AHEI and DASH dietary indices, the kidney stone group had a statistically lower dietary index, indicating that an unhealthy diet may increase the risk of kidney stones. For uric acid, the kidney stone patient was 5.67mg/dL, which was higher than the 5.43 mg/dL in the non-kidney stone group. In Supplementary Material (Table S1), four dietary indices’ mean, geometric mean and distributions were noted.

# 3.2 Association between dietary index and risk of kidney stones

To explore the association between individual dietary indices and the risk of kidney stones, we performed univariate logistic regression with adjustment for relevant confounders on the basis of the presence or absence of kidney stones. The results are shown in Table 2. After adjusting for confounding variables, the risk of kidney stones

decreased with increasing scores on all four dietary indices, with AHEI (OR:0.992, 95 % CI: 0.990-0.994), DASH (OR:0.976, 95 % CI: 0.970-0.991), HEI2020 (OR:0.989, 95 %

CI: 0.987-0.991), MED (OR:0.941, 95 % CI:0.904-0.979), all P < 0.05. This suggests

that a healthier diet is important for the prevention of kidney stones.

# 3.3 Non-linearity and threshold effect analysis between dietary index and risk of kidney stones

RCS curves were used to examine potential nonlinear associations in the relationship between the four dietary indices and the likelihood of kidney stones, and the results are shown in Figure 2. The overall associations between the four dietary indices (AHEI, DASH, HEI2020, and MED) and kidney stone risk were all statistically significant (all P-overall < 0.001). The HEI2020 and MED with the kidney stone risk shows an linear trend (P value for Nonlinear >0.05), while the AHEI and DASH with the kidney stone risk exhibit nonlinear trends (P value for Nonlinear <0.05). Of which, AHEI displayed an inverted U-shaped association with kidney stone risk, with the inflection point occurring at AHEI index scores: 37.73. DASH exhibited an inverted U- shaped association with kidney stone risk, with the inflection point occurring at DASH index scores: 21.96.

# 3.4 Association of mixed effects of dietary index with kidney stones evaluated by

**WQS**

We performed Pearson correlation analysis on the four dietary indices and found significant positive correlations among the four dietary indices (as shown in Figure 3A). Figure 3B show the results of the mixed effects of dietary components from various dietary indices on the risk of kidney stone within the WQS model. WQS regression results indicated that the mixed effects of the four dietary indices were negatively correlated with kidney stones, with the highest weight attributed to HEI2020 (49.2 %), followed by DASH (26.4 %), MED (21.8%), and AHEI (2.9 %).

# 3.5 BKMR model to assess the effect of dietary index on kidney stones

We used the BKMR model, which is a nonparametric Bayesian variable selection framework to assess the combined effect of multiple dietary indices on kidney stones. We initially assessed the univariate exposure-response associations between single

dietary index and kidney stones. Figure 3C illustrates a negative exposure-response trend for each dietary indices to the risk of kidney stones across the total population. The overall effect of the BKMR mixture assessment indicated that dietary indices levels at the 60th percentile and above were associated with significantly lower rates of kidney stones than exposure levels fixed at the 50th percentile (Figure 3D). Additionally, Figure 3E illustrates the single-variable effects of various dietary indices on the risk of kidney stones at the 25th, 50th, and 75th quantiles. HEI2020, DASH, MED, and AHEI are negative related with kidney stones at all three quantiles, indicating that higher levels of these dietary indices are correlated with a decreased risk of kidney stones.

The bivariate relationship was further explored by examining four dietary indices. Figure 4 presents the relationship between expos1 and the quantiles of expos2 for various dietary indices scores (HEI2020, DASH, MED, and AHEI). It shows the relationship between dietary indices in the column and kidney stones when the dietary index mixture in the row is fixed at its 25th, 50th, and 75th percentiles and the remaining dietary indices are fixed at its 50th percentile. There was a clear interaction between these dietary indices.

# 3.6 Intermediation effect analysis

To investigate whether uric acid mediates the associations among dietary indices and kidney stones, we examined the mediating role of uric acid in HEI2020, DASH, MED, and AHEI, which are significantly associated with kidney stones. The results are shown in Figure 5A-D, where uric acid mediated the relationship between the four dietary indices and kidney stones. The mediating proportions of AHEI, DASH, HEI2020 and MED were 12.14%, 5.88%, 2.52% and 25.56%, respectively. The P value of mediating effect of DASH was 0.062, and the rest were less than 0.05, indicating that healthy diet had a great impact on the risk of kidney stones, and there was a mediating effect mediated by uric acid. Among them, MED restricted the intake of fat and protein more strictly, and the mediating effect of uric acid was more prominent.

# 4. Discussion

This study investigated the association between the four dietary indices and the

risk of kidney stones and the mediating role of uric acid levels in the relationship between diet and kidney stones. By analyzing the data from the NHANES database, we found that the OR for kidney stones decreased significantly with increasing AHEI, DASH, HEI2020, MED. This negative relationship remained robust after adjusting for potential confounders such as age, sex, race, PIR, education level, marriage, BMI, smoking, alcohol consumption, DM, and hypertension. Specifically, AHEI and DASH were inversely associated with the risk of kidney stones only above 31.75 and 21.96, while HEI2020 and MED were inversely associated with the risk of kidney stones. In addition, blood uric acid levels mediated the four dietary indices and the risk of kidney stones.

Multiple analyses were performed for the separate effects of the four dietary indices on the risk of kidney stones. Logistic regression analysis showed that MED showed a lower OR value in Crude model, Model 1 and Model 2. Correlation analysis showed that there was a high correlation among the four dietary indices, and the WQS regression model could be used to explore the total effect of mixed exposure and the relative contribution of each factor. WQS weight showed that HEI2020 had the highest proportion weight of 0.492, that is, HEI2020 had the greatest impact on the risk of kidney stones. In the BKMR forest plot, HEI2020 indicated a lower risk of kidney stones when quartile detection of the index was performed. In conclusion, in separate effect analyses, the MED and HEI2020 indexes had the greatest impact on kidney stone risk.

The AHEI advocates the consumption of fruits, vegetables, grains, and vegetable

oils, while discouraging the consumption of sugar-sweetened beverages, alcohol, and meat [22]. AHEI was found to be significantly associated with gallstone risk reduction in previous studies [23], however, no study has explored the relationship between AHEI and kidney stones [22]. This study is the first to find that AHEI diet can inhibit the occurrence of kidney stones when it exceeds 31.75.

The DASH diet has been associated with a reduction in kidney stones in previous studies[24]. The DASH diet is associated with decreased calcium oxalate supersaturation, increased magnesium and citrate excretion, and increased urine pH

[25]. Higher DASH score was associated with higher urine potassium, magnesium, phosphate, and pH, and lower relative supersaturations (RSS) of calcium oxalate (women only) and uric acid[26]. The DASH diet reduced the incidence of stones by changing the characteristics of urine [27]. The DASH diet is characterized by a high intake of fruits, vegetables, whole grains, and low-fat dairy products, as well as a low intake of sodium, red and processed meats, and added sugars. The DASH recommends a reduction in sodium intake while emphasizing dairy intake to promote kidney health and is related to the intake of calcium and phosphorus [28]. The DASH diet, which restricts the intake of meat protein, may reduce stone formation to a large extent through the mediating effect of uric acid.

HEI2020 encourages high intake of vegetables, fruits, and low-fat dairy products, which help to reduce uric acid levels and increase urine pH. Limiting refined grains and foods with a high oxalate content may reduce the risk of calcium oxalate stones. Emphasizing a balanced intake of calcium and magnesium in the diet can help reduce the likelihood of stone formation. Diet may influence stone risk by affecting the gut microbiota and altering oxalate metabolism and absorption[29, 30]. This is consistent with the inhibition of stone formation by HEI2020 in this study.

The MED Mediterranean diet is rich in potassium, magnesium, and low in saturated fat, components that help to increase urine pH and reduce urinary calcium excretion, thereby reducing the risk of calcium oxalate and urate stones. A high- potassium dietary pattern is associated with a reduced risk of kidney stones, and fruits and vegetables in the Mediterranean diet are the main sources of potassium[31, 32]. Kidney stone formation may be associated with chronic inflammation and oxidative stress, and the antioxidant components such as polyphenols and anti-inflammatory mechanisms of the Mediterranean diet help reduce these risks[33].

Because the healthy eating index was generally consistent in terms of dietary types, there was a high correlation and interaction among the four diets in this study, and the combined effect of multiple healthy eating indexes was significantly higher in reducing the risk of kidney stones. The diversity of healthy dietary patterns may optimize the urinary metabolic environment through the combined intake of potassium, magnesium,

calcium, and antioxidant components. Reducing urinary calcium excretion (e.g., DASH), regulating urine pH (e.g., MED), and lowering uric acid levels (e.g., AHEI) may reduce stone risk through a combination of different pathways[34, 35]. A study based on the NHS in the United States showed that the DASH and MED diets reduced the incidence of kidney stones, respectively, and this protective effect may be achieved by regulating urinary metabolic components and reducing inflammation[36]. In addition, this study identified lower uric acid levels as a mediator between multiple healthy dietary states and the risk of kidney stones. Uric acid level is an important cause of renal uric acid stones and other types of stones[37]which is affected by healthy diet level[38]. The AHEI diet emphasizes reducing the consumption of sugar-sweetened beverages, a feature that is particularly important for controlling uric acid levels[39]. The DASH diet was associated with most metabolic parameters, negatively correlated with triglycerides, urinary sodium, and uric acid, and positively correlated with serum vitamin D[40, 41]. Olive oil and nuts of the MED diet are rich in polyphenolic antioxidants, which reduce uric acid production and related tissue damage by inhibiting inflammation and oxidative stress. Both DASH and MED diets contribute to weight management, improve insulin resistance, and indirectly reduce uric acid levels[42, 43].

Despite the significant findings of this study, several limitations should be

acknowledged. First, the cross-sectional nature of the analysis precludes establishing causal relationships. Future longitudinal studies are essential to confirm the observed associations and mediation effects. Second, dietary data were based on 24-hour recall interviews, which may introduce recall bias and fail to capture long-term dietary habits accurately. Third, although uric acid's mediating role was identified, the precise molecular mechanisms underlying this mediation remain unexplored and warrant further biological investigation. Fourth, while extensive covariate adjustments were performed, residual confounding due to unmeasured or unknown factors cannot be entirely ruled out. Lastly, the findings are based on a U.S. population sample and may not be generalizable to other ethnicities or regions. Future research should employ prospective study designs, incorporate comprehensive dietary assessments, and explore biological pathways to better understand the intricate relationships between diet, uric

acid, and kidney stone formation in diverse populations.

# 5. Conclusion

In conclusion, this study provides robust evidence linking healthy dietary patterns to reduced kidney stone risk. Four dietary indices, including MED, DASH, AHEI, and HEI-2020, were negatively associated with kidney stone prevalence. MED and HEI- 2020 had the strongest protective effects. Mediation analysis confirmed that uric acid partially mediates these associations, with MED showing the highest mediation proportion. These findings highlight the critical role of diet in kidney stone prevention and the potential mechanisms involving uric acid regulation.

# Authors' contributions

Conceptualization, Jinlong Cao; Data curation, Lingyu Guo; Formal analysis, Jinlong Cao; Funding acquisition, Feng Li; Investigation, Tianyuan Zhai; Methodology, Jinlong Cao; Project administration, Yue Chong; Resources, Qi Chen; Software, Qian Wang; Supervision, Delai Fu; Validation, Li Xue; Visualization, Delai Fu, Li Xue, and

Feng Li; Writing – original draft, Jinlong Cao; Writing – review & editing, Feng Li.

# Funding

This work was supported by the Natural Science Basic Research Program of Shaanxi Province (Grant No. 2020JQ-544), and the National Science Foundation for Young Scientists of China (Grant No. 82100812).

# Institutional Review Board Statement

The data from NHANES has been approved by the Institutional Review Board of the NCHS, and all participants provided written informed consent.

# Data Availability Statement

The used data and materials during the present study are available from the corresponding author on reasonable request.

# Conflicts of Interest

Authors declare no conflict of interests.

# Reference

1. Abufaraj, M.; Xu, T.; Cao, C.; Waldhoer, T.; Seitz, C.; D’andrea, D.; Siyam, A.; Tarawneh, R.; Fajkovic, H.; Schernhammer, E.; et al. Prevalence and Trends in Kidney Stone Among Adults in the USA: Analyses of National Health and Nutrition Examination Survey 2007–2018 Data. *European Urology Focus.* 2021, 7, 1468-1475.
2. Peerapen, P.; Thongboonkerd, V. Kidney Stone Prevention. *Advances in Nutrition.* 2023, 14, 555-

569.

3. Chewcharat, A.; Curhan, G. Trends in the prevalence of kidney stones in the United States from 2007 to 2016. *Urolithiasis.* 2021, 49, 27-39.
4. Romero, V.; Akpinar, H.; Assimos, D.G. Kidney stones: a global picture of prevalence, incidence, and associated risk factors. *Rev Urol.* 2010, 12, e86-96.
5. Alelign, T.; Petros, B. Kidney Stone Disease: An Update on Current Concepts. *Adv Urol.* 2018, 2018, 3068365.
6. Lee, J.A.; Abramowitz, M.K.; Kipperman, N.; Drzewiecki, B.A.; Melamed, M.L.; Stern, J.M. Exploring the Association of Asthma with Urinary Stone Disease: Results from the National Health and Nutrition Examination Survey 2007–2014. *European Urology Focus.* 2020, 6, 354-360.
7. Ticinesi, A.; Nouvenne, A.; Borghi, L.; Meschi, T. Water and other fluids in nephrolithiasis: State of the art and future challenges. *Crit Rev Food Sci Nutr.* 2017, 57, 963-974.
8. Abate, V.; Vergatti, A.; Iaccarino Idelson, P.; Recano, C.; Brancaccio, M.; Prezioso, D.; Muscariello, R.; Nuzzo, V.; De Filippo, G.; Strazzullo, P.; et al. Adherence to Mediterranean Diet, Dietary Salt Intake, and Susceptibility to Nephrolithiasis: A Case-Control Study. *Nutrients.* 2024, 16.
9. Littlejohns, T.J.; Neal, N.L.; Bradbury, K.E.; Heers, H.; Allen, N.E.; Turney, B.W. Fluid Intake and Dietary Factors and the Risk of Incident Kidney Stones in UK Biobank: A Population-based Prospective Cohort Study. *European Urology Focus.* 2020, 6, 752-761.
10. Li, J.; Wu, Z.; Xin, S.; Xu, Y.; Wang, F.; Liu, Y.; Wang, S.; Dong, Y.; Guo, Y.; Han, Y.; et al. Body mass index mediates the association between four dietary indices and phenotypic age acceleration in adults: a cross-sectional study. *Food Funct.* 2024, 15, 7828-7836.
11. Ma, G.; Zhang, S.; Luo, Y.; Zhang, C.; Xu, W.; Wang, L. The association between composite dietary antioxidant index and rheumatoid arthritis: evidence from NHANES 2001-2020. *BMC Rheumatol.* 2024, 8, 74.
12. Zhang, C.; Qiu, S.; Bian, H.; Tian, B.; Wang, H.; Tu, X.; Cai, B.; Jin, K.; Zheng, X.; Yang, L.; Wei,

Q. Association between Dietary Inflammatory Index and kidney stones in US adults: data from the National Health and Nutrition Examination Survey (NHANES) 2007–2016. *Public Health Nutrition.* 2021, 24, 6113-6121.

13. Maddahi, N.S.; Fotros, D.; Sohouli, M.H.; Mozaffari-Khosravi, H.; Khayyatzadeh, S.S. Higher dietary insulin index is directly associated with the odd of kidney stones. *Sci Rep.* 2024, 14, 28302.
14. Bai, S.; Zhang, Y.; Guo, C.; Liu, Y.; Zhang, Q.; Liu, L.; Sun, S.; Wang, X.; Zhou, M.; Jia, Q.; et al. Associations between dietary patterns and nephrolithiasis risk in a large Chinese cohort: is a balanced or plant-based diet better? *Food Funct.* 2023, 14, 3220-3229.
15. Normand, M.; Haymann, J.P.; Daudon, M. Medical treatment of uric acid kidney stones. *Can Urol Assoc J.* 2024, 18, E339-e345.
16. Zhan, J.J.; Hodge, R.A.; Dunlop, A.L.; Lee, M.M.; Bui, L.; Liang, D.; Ferranti, E.P. Dietaryindex: a user-friendly and versatile R package for standardizing dietary pattern analysis in epidemiological and clinical studies. *Am J Clin Nutr.* 2024, 120, 1165-1174.
17. Bernert, J.T.; Jacob, P., 3rd; Holiday, D.B.; Benowitz, N.L.; Sosnoff, C.S.; Doig, M.V.; Feyerabend, C.; Aldous, K.M.; Sharifi, M.; Kellogg, M.D.; Langman, L.J. Interlaboratory comparability of serum cotinine measurements at smoker and nonsmoker concentration levels: a round-robin study. *Nicotine Tob Res.* 2009, 11, 1458-1466.
18. Lei, T.; Li, M.; Zhu, Z.; Yang, J.; Hu, Y.; Hua, L. Comprehensive evaluation of serum cotinine on human health: Novel evidence for the systemic toxicity of tobacco smoke in the US general population. *Sci Total Environ.* 2023, 892, 164443.
19. Hicks, C.W.; Wang, D.; Matsushita, K.; Windham, B.G.; Selvin, E. Peripheral Neuropathy and All- Cause and Cardiovascular Mortality in U.S. Adults : A Prospective Cohort Study. *Ann Intern Med.* 2021, 174, 167-174.
20. Keil, A.P.; Buckley, J.P.; O'Brien, K.M.; Ferguson, K.K.; Zhao, S.; White, A.J. A Quantile-Based g- Computation Approach to Addressing the Effects of Exposure Mixtures. *Environ Health Perspect.* 2020, 128, 47004.
21. Bobb, J.F.; Valeri, L.; Claus Henn, B.; Christiani, D.C.; Wright, R.O.; Mazumdar, M.; Godleski, J.J.; Coull, B.A. Bayesian kernel machine regression for estimating the health effects of multi-pollutant mixtures. *Biostatistics.* 2015, 16, 493-508.
22. Fung, T.T.; Meyer, H.E.; Willett, W.C.; Feskanich, D. Association between Diet Quality Scores and Risk of Hip Fracture in Postmenopausal Women and Men Aged 50 Years and Older. *J Acad Nutr Diet.* 2018, 118, 2269-2279.e2264.
23. Wirth, J.; Song, M.; Fung, T.T.; Joshi, A.D.; Tabung, F.K.; Chan, A.T.; Weikert, C.; Leitzmann, M.; Willett, W.C.; Giovannucci, E.; Wu, K. Diet-quality scores and the risk of symptomatic gallstone disease: a prospective cohort study of male US health professionals. *Int J Epidemiol.* 2018, 47, 1938-1946.
24. Taylor, E.N.; Fung, T.T.; Curhan, G.C. DASH-style diet associates with reduced risk for kidney stones. *J Am Soc Nephrol.* 2009, 20, 2253-2259.
25. Noori, N.; Honarkar, E.; Goldfarb, D.S.; Kalantar-Zadeh, K.; Taheri, M.; Shakhssalim, N.; Parvin, M.; Basiri, A. Urinary lithogenic risk profile in recurrent stone formers with hyperoxaluria: a randomized controlled trial comparing DASH (Dietary Approaches to Stop Hypertension)-style and low-oxalate diets. *Am J Kidney Dis.* 2014, 63, 456-463.
26. Taylor, E.N.; Stampfer, M.J.; Mount, D.B.; Curhan, G.C. DASH-style diet and 24-hour urine

composition. *Clin J Am Soc Nephrol.* 2010, 5, 2315-2322.

27. Rodrigues, F.G.; Lima, T.M.; Zambrano, L.; Heilberg, I.P. Dietary pattern analysis among stone formers: resemblance to a DASH-style diet. *J Bras Nefrol.* 2020, 42, 338-348.
28. Cena, H.; Calder, P.C. Defining a Healthy Diet: Evidence for The Role of Contemporary Dietary Patterns in Health and Disease. *Nutrients.* 2020, 12.
29. Batagello, C.A.; Monga, M.; Miller, A.W. Calcium Oxalate Urolithiasis: A Case of Missing Microbes? *J Endourol.* 2018, 32, 995-1005.
30. Crivelli, J.J.; Mitchell, T.; Knight, J.; Wood, K.D.; Assimos, D.G.; Holmes, R.P.; Fargue, S. Contribution of Dietary Oxalate and Oxalate Precursors to Urinary Oxalate Excretion. *Nutrients.* 2020, 13.
31. Sorensen, M.D.; Kahn, A.J.; Reiner, A.P.; Tseng, T.Y.; Shikany, J.M.; Wallace, R.B.; Chi, T.; Wactawski-Wende, J.; Jackson, R.D.; O'Sullivan, M.J.; et al. Impact of nutritional factors on incident kidney stone formation: a report from the WHI OS. *J Urol.* 2012, 187, 1645-1649.
32. Weaver, C.M. Potassium and health. *Adv Nutr.* 2013, 4, 368s-377s.
33. Çıtar Dazıroğlu, M.E.; Acar Tek, N. The Effect on Inflammation of Adherence to the Mediterranean Diet in Polycystic Ovary Syndrome. *Curr Nutr Rep.* 2023, 12, 191-202.
34. Ferraro, P.M.; Bargagli, M.; Trinchieri, A.; Gambaro, G. Risk of Kidney Stones: Influence of Dietary Factors, Dietary Patterns, and Vegetarian-Vegan Diets. *Nutrients.* 2020, 12.
35. Wang, Y.; Liu, S.; Zhao, Q.; Wang, N.; Liu, X.; Zhang, T.; He, G.; Zhao, G.; Jiang, Y.; Chen, B. Analysis of Dietary Patterns Associated with Kidney Stone Disease Based on Data-Driven Approaches: A Case-Control Study in Shanghai. *Nutrients.* 2024, 16.
36. Littlejohns, T.J.; Neal, N.L.; Bradbury, K.E.; Heers, H.; Allen, N.E.; Turney, B.W. Fluid Intake and Dietary Factors and the Risk of Incident Kidney Stones in UK Biobank: A Population-based Prospective Cohort Study. *Eur Urol Focus.* 2020, 6, 752-761.
37. Ferraro, P.M.; Curhan, G.C. Serum Uric Acid and Risk of Kidney Stones. *Am J Kidney Dis.* 2017, 70, 158-159.
38. Cheng, S.; Shan, L.; You, Z.; Xia, Y.; Zhao, Y.; Zhang, H.; Zhao, Z. Dietary patterns, uric acid levels, and hyperuricemia: a systematic review and meta-analysis. *Food Funct.* 2023, 14, 7853-7868.
39. Choi, H.K.; Curhan, G. Soft drinks, fructose consumption, and the risk of gout in men: prospective cohort study. *Bmj.* 2008, 336, 309-312.
40. Vahid, F.; Hoge, A.; Hébert, J.R.; Bohn, T. Association of diet quality indices with serum and metabolic biomarkers in participants of the ORISCAV-LUX-2 study. *Eur J Nutr.* 2023, 62, 2063-2085.
41. Heindel, J.; Baid-Agrawal, S.; Rebholz, C.M.; Nadal, J.; Schmid, M.; Schaeffner, E.; Schneider, M.P.; Meiselbach, H.; Kaesler, N.; Bergmann, M.; et al. Association Between Dietary Patterns and Kidney Function in Patients With Chronic Kidney Disease: A Cross-Sectional Analysis of the German Chronic Kidney Disease Study. *J Ren Nutr.* 2020, 30, 296-304.
42. Juraschek, S.P.; Gelber, A.C.; Choi, H.K.; Appel, L.J.; Miller, E.R., 3rd. Effects of the Dietary Approaches to Stop Hypertension (DASH) Diet and Sodium Intake on Serum Uric Acid. *Arthritis Rheumatol.* 2016, 68, 3002-3009.
43. Angelico, F.; Baratta, F.; Coronati, M.; Ferro, D.; Del Ben, M. Diet and metabolic syndrome: a narrative review. *Intern Emerg Med.* 2023, 18, 1007-1017.

# Figure legend

Figure 1. Flowchart of the study.

Figure 2. The exposure-response association of the dietary indices with the kidney stone by restricted cubic spline (RCS). (A) Nonlinear association between AHEI index and the risk of kidney stone. (B) Nonlinear association between DASH index and the risk of kidney stone. (C) Nonlinear association between HEI2020 index and the risk of kidney stone. (D) Nonlinear association between MED index and the risk of kidney stone.

Figure 3. Mixed effects and total effects of four dietary indices on kidney stone. (A) Spearman correlations among the MED, DASH, AHEI, and HEI-2020. (B) Mixed effects of the four dietary indices on kidney stone assessed by WQS. (C) Univariate exposure–response function and 95% CI scores for the associations between single dietary indices exposures when other dietary indices exposures are fixed at the median level. (D) Summary of the overall health effects of the combined exposures on the four dietary indices at various quantiles (from 25th to 75th) by BKMR. (E) Single-variable effects of dietary indices at decreasing quartiles for the kidney stone.

Figure 4. Bivariate exposure–response functions of dietary indices with the kidney stone.

Figure 5. Uric acid mediates the associations between dietary indices and the risk of kidney stone. (A) Partial mediating effect of uric acid on the association between AHEI and the risk of kidney stone. (B) Partial mediating effect of uric acid on the association between DASH and the risk of kidney stone. (C) Partial mediating effect of uric acid on the association between HEI2020 and the risk of kidney stone. (D) Partial mediating effect of uric acid on the association between MED and the risk of kidney stone.

Figure 1	[Click here to access/download;Figure;Figure 1.tif](https://www2.cloud.editorialmanager.com/pone/download.aspx?id=41407793&guid=5e315e53-0d85-48d0-9d6a-49d510f8432c&scheme=1)[<https://www2.cloud.editorialmanager.com/pone/download.aspx?id=41407793&guid=5e315e53-0d85-48d0-9d6a-49d510f8432c&scheme=1>](https://www2.cloud.editorialmanager.com/pone/download.aspx?id=41407793&guid=5e315e53-0d85-48d0-9d6a-49d510f8432c&scheme=1)

Figure 2	[Click here to access/download;Figure;Figure 2.tif](https://www2.cloud.editorialmanager.com/pone/download.aspx?id=41407799&guid=507de34f-0e02-47f3-a1e8-4c1770b65df6&scheme=1)[<https://www2.cloud.editorialmanager.com/pone/download.aspx?id=41407799&guid=507de34f-0e02-47f3-a1e8-4c1770b65df6&scheme=1>](https://www2.cloud.editorialmanager.com/pone/download.aspx?id=41407799&guid=507de34f-0e02-47f3-a1e8-4c1770b65df6&scheme=1)

Figure 3	[Click here to access/download;Figure;Figure 3.tif](https://www2.cloud.editorialmanager.com/pone/download.aspx?id=41407804&guid=c23a9bc4-606e-4664-a7cf-019570a11c42&scheme=1)[<https://www2.cloud.editorialmanager.com/pone/download.aspx?id=41407804&guid=c23a9bc4-606e-4664-a7cf-019570a11c42&scheme=1>](https://www2.cloud.editorialmanager.com/pone/download.aspx?id=41407804&guid=c23a9bc4-606e-4664-a7cf-019570a11c42&scheme=1)

Figure 4	[Click here to access/download;Figure;Figure 4.tif](https://www2.cloud.editorialmanager.com/pone/download.aspx?id=41407865&guid=747f0ad6-40ee-4d8c-a4c6-8103140d6296&scheme=1)[<https://www2.cloud.editorialmanager.com/pone/download.aspx?id=41407865&guid=747f0ad6-40ee-4d8c-a4c6-8103140d6296&scheme=1>](https://www2.cloud.editorialmanager.com/pone/download.aspx?id=41407865&guid=747f0ad6-40ee-4d8c-a4c6-8103140d6296&scheme=1)

Figure 5	[Click here to access/download;Figure;Figure 5.tif](https://www2.cloud.editorialmanager.com/pone/download.aspx?id=41407867&guid=ad5bd5ba-c14f-4bb9-9036-3b6c64eb9fde&scheme=1)[<https://www2.cloud.editorialmanager.com/pone/download.aspx?id=41407867&guid=ad5bd5ba-c14f-4bb9-9036-3b6c64eb9fde&scheme=1>](https://www2.cloud.editorialmanager.com/pone/download.aspx?id=41407867&guid=ad5bd5ba-c14f-4bb9-9036-3b6c64eb9fde&scheme=1)

Table 1	[Click here to access/download;Figure;Table 1.docx](https://www2.cloud.editorialmanager.com/pone/download.aspx?id=41407892&guid=b299786a-2a86-47a9-a0c1-1ed695e76b32&scheme=1)[<https://www2.cloud.editorialmanager.com/pone/download.aspx?id=41407892&guid=b299786a-2a86-47a9-a0c1-1ed695e76b32&scheme=1>](https://www2.cloud.editorialmanager.com/pone/download.aspx?id=41407892&guid=b299786a-2a86-47a9-a0c1-1ed695e76b32&scheme=1)

Table 1. Baseline characteristics of included participants.

| Characteristics | Overall | Non-kidney stone | Kidney stone | P |
| --- | --- | --- | --- | --- |
| Number | 25421 | 22951 | 2470 |  |
| Age (Years) | 50.26 ± 17.52 | 49.58 ± 17.56 | 56.50 ± 15.87 | <0.001 |
| Gender |  |  |  | <0.001 |
| Female | 11034 (52.5) | 10127 (53.5) | 907 (43.8) |  |
| Male | 9967 (47.5) | 8802 (46.5) | 1165 (56.2) |  |
| BMI (kg/m2) | 29.48 ± 7.01 | 29.36 ± 7.02 | 30.52 ± 6.77 | <0.001 |
| Race |  |  |  | <0.001 |
| Mexican American | 3317 (15.7%) | 3055 (16.0%) | 262 (12.5%) |  |
| Non-Hispanic Black | 4192 (19.8%) | 3933 (20.6%) | 259 (12.4%) |  |
| Non-Hispanic White | 9317 (44.0%) | 8110 (42.5%) | 1207 (57.8%) |  |
| Other Hispanic | 2211 (10.4%) | 1996 (10.5%) | 215 (10.3%) |  |
| Other Race | 2126 (10.0%) | 1981 (10.4%) | 145 (6.9%) |  |
| Education |  |  |  | 0.022 |
| Less Than 9th Grade | 1997 (9.4%) | 1804 (9.5%) | 193 (9.2%) |  |
| 9-11th Grade | 2841 (13.4%) | 2549 (13.4%) | 292 (14.0%) |  |
| High School Grad | 4906 (23.2%) | 4431 (23.2%) | 475 (22.7%) |  |
| Some College or AA | 6338 (29.9%) | 5654 (29.6%) | 684 (32.8%) |  |
| College Graduate or | 5060 (23.9%) | 4617 (24.2%) | 443 (21.2%) |  |
| Refused | 6 (0.0%) | 6 (0.0%) | 0 (0.0%) |  |
| Missing | 15 (0.1%) | 14 (0.1%) | 1 (0.0%) |  |
| Marital Status |  |  |  | <0.001 |
| Married | 11177 (52.8%) | 9961 (52.2%) | 1216 (58.2%) |  |
| Living with partner | 1688 (8.0%) | 1562 (8.2%) | 126 (6.0%) |  |
| Divorced | 2364 (11.2%) | 2075 (10.9%) | 289 (13.8%) |  |
| Separated | 695 (3.3%) | 630 (3.3%) | 65 (3.1%) |  |
| Never married | 3601 (17.0%) | 3405 (17.9%) | 196 (9.4%) |  |
| Widowed | 1631 (7.7%) | 1436 (7.5%) | 195 (9.3%) |  |
| Refused | 6 (0.0%) | 5 (0.0%) | 1 (0.0%) |  |
| Missing | 1 (0.0%) | 1 (0.0%) | 0 (0.0%) |  |
| HBP |  |  |  | <0.001 |
| No | 13360 (63.1%) | 12351 (64.7%) | 1009 (48.3%) |  |
| Yes | 7779 (36.8%) | 6702 (35.1%) | 1077 (51.6%) |  |
| Missing | 24 (0.1%) | 22 (0.1%) | 2 (0.1%) |  |
| Diabetes |  |  |  | <0.001 |
| No | 17823 (84.2%) | 16276 (85.3%) | 1547 (74.1%) |  |
| Borderline | 520 (2.5%) | 454 (2.4%) | 66 (3.2%) |  |
| Yes | 2809 (13.3%) | 2336 (12.2%) | 473 (22.7%) |  |
| Missing | 11 (0.1%) | 9 (0.0%) | 2 (0.1%) |  |
| Cotinine (ng/mL) | 55.57 ± 127.20 | 54.49 ± 125.57 | 65.39 ± 140.91 | <0.001 |
| Alcohol |  |  |  | <0.001 |
| Never | 4174 (19.9%) | 3771 (19.9%) | 403 (19.4%) |  |
| Former | 1020 (4.9%) | 911 (4.8%) | 109 (5.3%) |  |
| Mild | 7900 (37.6%) | 7040 (37.2%) | 860 (41.5%) |  |
| Moderate | 3673 (17.5%) | 3334 (17.6%) | 339 (16.4%) |  |
| Heavy | 4234 (20.2%) | 3873 (20.5%) | 361 (17.4%) |  |
| MED | 3.47 ± 1.37 | 3.48 ± 1.37 | 3.37 ± 1.34 | <0.001 |
| HEI2020 | 51.52 ± 12.04 | 51.64 ± 12.09 | 50.37 ± 11.54 | <0.001 |
| AHEI | 38.53 ± 11.49 | 38.59 ± 11.53 | 38.02 ± 11.07 | 0.032 |
| DASH | 22.37 ± 5.01 | 22.40 ± 5.04 | 22.08 ± 4.80 | 0.005 |
| Uric acid (mg/dL) | 5.45 ± 1.45 | 5.43 ± 1.44 | 5.67 ± 1.52 | <0.001 |

Mean ± SD for continuous variables, % for categorical variables

BMI, body mass index; HBP, hypertension; MED, Mediterranean Diet; HEI2020, Healthy Eating Index-2020; AHEI, Alternate Healthy Eating Index; DASH, Dietary Approaches to Stop Hypertension

Table 2	[Click here to access/download;Figure;Table 2.docx](https://www2.cloud.editorialmanager.com/pone/download.aspx?id=41407893&guid=aa9bfc83-082f-4436-ac8f-2cf27ec037cb&scheme=1)[<https://www2.cloud.editorialmanager.com/pone/download.aspx?id=41407893&guid=aa9bfc83-082f-4436-ac8f-2cf27ec037cb&scheme=1>](https://www2.cloud.editorialmanager.com/pone/download.aspx?id=41407893&guid=aa9bfc83-082f-4436-ac8f-2cf27ec037cb&scheme=1)

Table 2 Multiple logistic regression analysis of the relationship between four dietary indices and kidney stones.

| Variable | Crude model | Model 1 |  | Model 2 |  |
| --- | --- | --- | --- | --- | --- |
|  | OR [95% CI] p-value | OR [95% CI] | p-value | OR [95% CI] | p-value |
| AHEI | 0.995 (0.993, 0.997) <0.001 | 0.988 (0.986, 0.990) | 0.001 | 0.992 (0.990, 0.994) | 0.003 |
| DASH | 0.985 (0.975, 0.995) <0.001 | 0.965 (0.957, 0.974) | <0.001 | 0.976 (0.970, 0.982) | <0.001 |
| HEI2020 | 0.990 (0.988, 0.992) <0.001 | 0.984 (0.982, 0.986) | <0.001 | 0.989 (0.987, 0.991) | <0.001 |
| MED | 0.929 (0.863, 0.999) <0.001 | 0.904 (0.870, 0.939) | <0.001 | 0.941 (0.904, 0.979) | 0.001 |

Crude mode: no covariates were adjusted.

Model 1: sex, age, race, educational level, and marital status were adjusted.

Model 2: model 1 + BMI, Cotinine, Alcohol drink, Diabetes and Hypertension (HBP) were fully adjusted.

Table s1

[Click here to access/download](https://www2.cloud.editorialmanager.com/pone/download.aspx?id=41407894&guid=07589c33-a627-40ee-91ca-29fc4c6a6108&scheme=1)

[**Supporting Information** Table S1.docx](https://www2.cloud.editorialmanager.com/pone/download.aspx?id=41407894&guid=07589c33-a627-40ee-91ca-29fc4c6a6108&scheme=1)
