# Supplementary material for: Uric acid levels mediate the association between four dietary indices and kidney stones in US adults: A cross-sectional study of NHANES 2007–2018
Source: PLoS One. 2026 Jan 2;21(1):e0339839. doi: 10.1371/journal.pone.0339839 (PMC12758690; doi:10.1371/journal.pone.0339839)
Supplement: Attachment — Submitted filename: Response_to_Reviewers_auresp_2.docx [file pone.0339839.s006.docx]

Dear Editors and Reviewers:

Thank you for your letter and for the reviewers’ comments concerning our manuscript entitled “Uric acid levels mediate the association between four dietary indices and kidney stones in US adults: a cross-sectional study of NHANES 2007-2018” (ID: PONE-D-25-41276). Those comments are all valuable and very helpful for revising and improving our manuscript, as well as the important guiding significance to our researches. We have studied comments carefully and have made correction which we hope meet with approval. Revised portion are marked in red in the paper. The main corrections in the paper and the responds to the reviewer’s comments are as flowing:

Responds to the reviewer’s comments:

**Reviewer #3:**

**Comment 1:** Please provide different age groups and BMI group range (ex: age: 20-30, 30-40, 40-50,50-60,60-70 or < 50 and > 50 (and reason behind that) BMI: < 18.5, 18.5 - 24.9, 25 - 29.9, 30-39.9, and > 40), instead of only stating the range and mean number. Providing age groups (not only stating the overall age range), might help enhance the discussion since it affects the BMI and might affect the overall result of this study. Thus, necessary to be stated and discussed.

**Response:** Thank you very much for your valuable comment. We apologize for the misunderstanding in our previous revision. In the updated manuscript, we have revised the demographic data presentation according to your suggestion. Specifically, age has now been categorized into two groups (<60 years and ≥60 years) based on widely accepted WHO criteria for defining older adults, which may better reflect differences related to the elderly status. In addition, BMI has been regrouped following WHO standards into <18.5 (underweight), 18.5–25 (normal weight), 25–30 (overweight), and ≥30 (obesity). These subgroup classifications have been incorporated into both the Results and Discussion sections to better explore their potential influence on BMI-related outcomes and overall study findings.

In addition, the editor's summary of the reviewers' opinions and the overall evaluation of the article are also very helpful, and we have followed the suggestions to revise it one by one.

In all, I found the reviewer’ s comments and the editor's summary are quite helpful, and I revised my paper point-by-point. Thank you and the review again for your help! We tried our best to improve the manuscript and made some changes in the manuscript. These changes will not influence the content and framework of the paper. And here we did not list the changes but marked in red in revised paper.

We appreciate for Editors/Reviewer’ warm work earnestly, and hope that the revision will meet with approval. Once again, thank you very much for your comments and suggestions.
